# Validation and clinical application of the Metacognitions Questionnaire in a sample of Brazilian generalized anxiety disorder patients: the effects of different treatment interventions

**DOI:** 10.47626/2237-6089-2021-0444

**Published:** 2023-08-04

**Authors:** Julia Karl Schwinn, Sofia Giusti Alves, Marianna de Abreu Costa, Francine Gonçalves, Carolina Blaya Dreher, Gisele Gus Manfro

**Affiliations:** 1 Universidade Federal do Rio Grande do Sul Hospital de Clínicas de Porto Alegre Porto Alegre RS Brazil Universidade Federal do Rio Grande do Sul (UFRGS), Hospital de Clínicas de Porto Alegre (HCPA), Programa de Transtornos de Ansiedade (PROTAN), Porto Alegre, RS, Brazil.; 2 UFRGS HCPA Porto Alegre RS Brazil UFRGS, HCPA, Programa de Pós-Graduação em Psiquiatria e Ciências do Comportamento, Porto Alegre, RS, Brazil.

**Keywords:** generalized anxiety disorder, metacognition, MCQ-30, mindfulness-based interventions, Body in Mind Training, selective serotonin reuptake inhibitor

## Abstract

**Introduction:**

Metacognitive beliefs about worry may trigger anxiety. However, the effect of generalized anxiety disorder (GAD) treatment on metacognition has not yet been investigated.

**Objectives:**

To validate the Metacognitions Questionnaire (MCQ-30) in a Brazilian GAD sample and verify whether different interventions reduce metacognitive beliefs.

**Method:**

We recruited 180 GAD individuals and randomized them to Body in Mind Training (BMT), Fluoxetine (FLX), or an active control group (Quality of Life [QoL]) for 8 weeks. The MCQ-30 was assessed for internal consistency, was evaluated with confirmatory and exploratory factor analyses, and was tested for convergent validity with the Penn State Worry Questionnaire (PSWQ). Generalized estimating equations (GEE) were employed to analyze differences after the interventions.

**Results:**

The MCQ-30 demonstrated good internal consistency and acceptability; the original five-factor model was supported. There was a positive moderate correlation between MCQ-30 scores and worry. GEE showed a significant group x time interaction (p < 0.001). Both BMT (mean difference [MD] = -6.04, standard error [SE] = -2.39, p = 0.034) and FLX (MD = -5.78, SE = 1.91, p = 0.007) reduced MCQ-30 scores. FLX was superior to QoL, but not BMT, at weeks 5 and 8. There were no differences between BMT and QoL.

**Conclusion:**

The Brazilian-Portuguese version of MCQ-30 showed good psychometric properties. Furthermore, the positive effect of FLX and BMT on metacognition suggests it may represent a potential therapeutic target.

## Introduction

Generalized anxiety disorder (GAD) is characterized by excessive anxiety and worry about several daily events and activities,^1^ resulting in significant functional impairment.^2^ Many cognitive models have been proposed to try to understand possible GAD mechanisms,^3^ raising the idea that worry metacognition plays a major role in GAD maintenance.^4 , 5^ According to Wells,^5^ individuals with GAD exhibit two distinct types of worry: type I, related to positive beliefs about worry, and type II, which represent negative beliefs about worry.^6^ In a process called meta-worry, type II worry leads to dysfunctional strategies to avoid anxiety and triggers worries about lack of mental control.^6^ These unsuccessful attempts to control thoughts might influence anxiety and emotional distress.^7^ Studies showed a positive correlation between elevated metacognitive beliefs and anxious symptoms.^8 , 9^ Furthermore, in a 7-month cohort, Ryum et al.^10^ described that both metacognitive beliefs and worry were independent predictors of development of anxiety symptoms. These findings are in accordance with the hypothesis that metacognition plays a major role in GAD. Negative beliefs about worry represent the core domain of metacognition in patients with GAD^11^ and can distinguish GAD from other anxiety disorders.^8 , 11^

Understanding the mechanisms associated with pathological worry may contribute to advances in the treatment of GAD.^11^ Many treatment modalities, such as pharmacotherapy and different types of psychotherapy interventions, are able to reduce anxiety symptoms.^12^ To date, however, few studies have analyzed whether these interventions are able to change different cognitive processes associated with GAD. Wells proposed that a state known as detached mindfulness might reduce anxiety.^13^ This approach is in agreement with some of the concepts that comprise mindfulness-based interventions (MBIs),^13^ such as promotion of acting with awareness and non-judging of inner experience.^14^ Therefore, MBIs that have been associated with improvement in participants’ anxious symptoms^15 , 16^ might induce more adaptive metacognitive beliefs in patients with GAD.

This study aimed to validate the short form of the Metacognitions Questionnaire (MCQ-30) in a Brazilian population with GAD, since there are no validation studies of the MCQ in Brazilian samples and metacognition is a concept that is very relevant to better understanding of GAD. Moreover, we aimed to assess whether pharmacotherapy and Body in Mind Training (BMT), a modality of MBI, would be able to reduce metacognitive beliefs when compared to an active control group in GAD patients who underwent a randomized clinical trial.

## Methods

### Participants

This is a three-arm randomized clinical trial conducted between 2016 and 2018 at the Hospital de Clínicas de Porto Alegre, a teaching hospital in the South of Brazil.^17^ We recruited the participants from the community through media advertisements, inviting individuals with GAD symptoms to participate in the study. Patients were screened by phone call with a brief measure for assessing GAD on a 7-item scale (Generalized Anxiety Disorder 7-item [GAD-7]) and then evaluated by trained clinicians. The inclusion criteria were (1) adults, with (2) a diagnosis of GAD by psychologists or psychiatrists through clinical evaluation with the Mini International Neuropsychiatric Interview (MINI) adapted for the Diagnostic and Statistical Manual of Mental Disorders, 5th edition (DSM-5),^1^ (3) availability to attend weekly 2-hour sessions for eight weeks, and (4) no current pharmacological or psychological treatment for GAD. Participants were excluded if they had lifetime bipolar or psychotic disorder, current eating or antisocial personality disorder, substance use disorder (except tobacco) or suicidal ideation in the last 6 months, a score greater than or equal to 23 on the Hamilton Depression Rating Scale (HAM-D), or contraindications to use of fluoxetine (FLX) or to application of MBIs or were pregnant or breastfeeding.

Three hundred and forty-two subjects were assessed for eligibility and 249 were randomized to the clinical trial. One hundred and eighty fully completed the MCQ-30 at baseline and were included in this analysis (61 participants in the BMT group, 62 in the FLX group, and 57 in the Quality of Life [QoL] group). Participants answered the rating scales at baseline, in the 5th week, and at the end of the trial (8th week). For more information about sample selection, see the original study by Costa et al.^17^

### Ethical statement

The ethical review board of Hospital de Clínicas de Porto Alegre approved the study (number 20160301) and all participants signed written informed consent prior to inclusion.

### Interventions

The BMT protocol is a mindfulness-based group intervention developed by Russell et al.^18^ that aims to improve attentional and emotional regulation. This protocol applies psychology, mindfulness features, and tai chi techniques to promote a self-compassionate and non-judgmental awareness of the present moment. This intervention consisted of 2-hour sessions delivered weekly for 8 consecutive weeks by a psychologist with formal training in the BMT protocol.

The pharmacological intervention consisted of 8 weeks of selective serotonin reuptake inhibitor treatment (FLX). An experienced psychiatrist evaluated the participants individually. The initial dose was 20 mg and the medication was increased up to a maximum dose of 60 mg, according to improvement in Hamilton Anxiety Scale (HAM-A) scores, tolerability, and patient acceptance. Blister packs were counted to assess medication compliance.

We used a QoL group as an active control for the BMT intervention. This group-intervention focused on anxiety psychoeducation and developing healthy habits, such as maintaining a healthy diet and exercising. The intervention was designed and applied by a psychologist. Two-hour sessions were delivered weekly for 8 weeks, covering the following subjects: (1) psychoeducation about GAD; (2) substance use; (3) sleep hygiene; (4) physical activity; and (5) healthy eating. Sessions 6, 7, and 8 focused on maintenance of the new healthy habits adopted by the participants.

### Instruments

#### Penn State Worry Questionnaire (PSWQ)

The PSWQ is a self-report measure of trait worry that comprises 16 items and was designed to measure pathological worry, covering several aspects of worry such as uncontrollability and excessiveness.^19^ Items are rated on a 5-point Likert scale (1 = not typical of me; 5 = very typical of me). Scores range from 16 to 80 and higher scores indicate higher levels of pathological worry. The PSWQ has been shown to have good psychometric properties and is able to discriminate GAD from other anxiety disorders.^20^ This scale has been validated for Brazilian samples with adequate internal consistency.^21^

#### MCQ-30 scale

The MCQ-30 scale is a short form of the MCQ.^22^ It is composed of five factors, which represent different domains of metacognition. Higher scores on the MCQ-30 and its factors indicate higher levels of unhelpful and maladaptive metacognition. Total scores range from 30 to 120 and factor scores range from 6 to 24. The items are rated using a categorical scale that ranges from 1 (do not agree) to 4 (agree very much). The five factors are: (1) cognitive confidence (e.g., “my memory can mislead me at times”); (2) positive beliefs about worry (e.g., “worrying helps me cope”); (3) cognitive self-consciousness (e.g., “I constantly examine my thoughts”); (4) negative beliefs about uncontrollability of thoughts and danger (e.g., “my worrying thoughts persist, no matter how I try to stop them”); and (5) beliefs about the need to control thoughts (e.g., “if I could not control my thoughts, I would not be able to function”).

## Adaptation of the MCQ-30 scale

The original author of the MCQ-30 scale^22^ gave us permission to translate this instrument into Brazilian Portuguese and validate it. We used the forward-backward procedure to translate and adapt the questionnaire for Brazilian Portuguese speakers to ensure conceptual maintenance.^23^ Two independent native Brazilian Portuguese translators proficient in English translated the MCQ-30 into Brazilian Portuguese and reached a consensus version. Afterwards, an English-speaking translator who was fluent in Brazilian Portuguese back translated the Brazilian Portuguese version into an English version that was accepted by the original author of the instrument. We found no significant changes in item meanings. We also asked an expert on metacognition concepts and a Portuguese speaker to evaluate the final version of the scale.

## Statistical analysis

We used chi-square for categorical variables and analysis of variance (ANOVA) for continuous variables with normal distributions or non-parametric tests for continuous variables with non-normal distributions to assess differences in baseline characteristics. A two-sided p-value of 0.05 or less was considered statistically significant. We conducted all analyses using IBM SPSS Statistics v.18 (IBM Corp. Released 2017), except for the goodness-of-fit analyses, which were performed using R v.3.5.1.

### Scale validation

We performed an exploratory factor analysis (EFA) using Promax rotation to model the factors of the Brazilian Portuguese version of the MCQ-30. We measured the suitability of our data for factor analyses using the Kaiser-Meyer-Olkin (KMO) test. A KMO value > 0.6 is considered acceptable.^24^ Instrument reliability was assessed using Cronbach’s alpha coefficient, which evaluates internal consistency.^25^ We considered a coefficient greater than 0.7 to be acceptable.^26^ Reliability was also assessed with the Spearman-Brown coefficient using the split-half test.

In order to evaluate construct validity, we used confirmatory factor analyses to determine the goodness-of-fit of the MCQ-30 items to a five-factor model, as obtained in the original MCQ-30 study.^22^ We assessed goodness-of-fit using the chi-square test, root mean squared error of approximation (RMSEA), comparative fix index (CFI), and Tucker-Lewis Index (TLI). The chi-square test is sensitive to even small differences in model fit and it is usually statistically significant in scale validations, implying that the specific factor model does not fit the data, which is why we used additional indexes to evaluate the goodness-of-fit. Conventional cutoff values were considered for fit^27^ : RMSEA values < 0.10 indicate acceptable fit; CFI and TLI values > 0.90 indicate acceptable fit. Convergent validity was assessed with Pearson coefficients for the correlations between the MCQ-30 total score and factors and the PSWQ scale. Correlations were predefined as small (< 0.3), moderate (0.3-0.5), or large (> 0.5).^28^

### Efficacy analysis

We conducted Generalized Estimating Equations (GEE) according to the intention-to-treat principle to investigate changes in the MCQ-30 and its five subscales over the eight weeks of intervention. When the effect of the GEE model was significant, the Bonferroni test for multiple comparisons was performed. We controlled the analysis for potential confounders between the intervention groups.

### Correlation analysis

We conducted a correlation analysis as a secondary analysis to verify any associations between reduction of metacognitive beliefs and improvement in worry levels. The difference in worry levels from baseline to the endpoint of the intervention was measured through the mean difference in PSWQ scores from baseline to the endpoint, while delta metacognition was measured through the mean difference in MCQ-30 scores from baseline to the endpoint. We used Pearson’s correlation coefficient and Spearman’s rho to compare variables with symmetrical and asymmetrical distributions respectively.^28^

## Results

One hundred and eighty individuals diagnosed with GAD were included in our sample. Most of the participants were female (72.8%). The educational level of our sample was high: 31.7% had incomplete higher education, 25% had completed high school, 26.2% had a university degree, and 13.4% had taken postgraduate courses. We used the criteria adopted by the Brazilian Institute of Geography and Statistics (IBGE)^29^ to evaluate socioeconomic level: 44.4 and 42.8% of the participants were categorized in economic classes B and C respectively.

The mean MCQ-30 score at baseline was 77.01 (standard deviation [SD] = 14.24) and the highest factor score observed was for the negative beliefs factor (mean = 18.18, SD = 4.18). The mean cognitive confidence score was 15.72 (SD = 5.37), the mean positive beliefs score was 12.33 (SD = 4.52), the mean cognitive self-consciousness score was 15.53 (SD = 3.82), and the mean beliefs about the need to control thoughts score was 15.13 (SD = 3.72). The mean total PSWQ score at baseline was 61.65 (SD = 7.72). Table 1 depicts the baseline demographic characteristics of the participants in each intervention group. Obsessive-compulsive disorder (OCD) diagnosis was the only variable with a statistically significant difference in the comparison between groups.

**Table 1:** Baseline characteristics of the sample

	BMT (n = 61)	FLX (n = 62)	QoL (n = 57)	p-value *
Age, years (mean [SD])	36.93 (13.61)	35.04 (11.38)	36.54 (12.72)	0.739
Female, %	73.3	83.9	81.0	0.332
Education, %				
Incomplete high school	5.1	0.0	0.0	0.312
Complete high school	0.0	3.2	1.8	
Complete higher education	59.3	61.3	56.1	
Postgraduate courses	35.6	35.5	42.1	
Economic class (%)				0.851
A	3.3	0.0	0.0	
B	46.7	50.0	50.0	
C	46.7	46.8	44.8	
D	0.0	0.0	0.6	
Unknown	3.3	3.2	3.4	
Axis I diagnosis, %				
Major depression	38.3	25.8	36.2	0.291
Panic disorder	33.3	25.0	41.7	0.476
Agoraphobia	11.7	6.5	20.7	0.062
Obsessive-compulsive disorder	10.0	0.0	3.4	**0.025**
Social anxiety disorder	10.0	6.5	15.5	0.210
Posttraumatic stress disorder	5.0	6.5	1.7	0.538
HAMA, (mean [SD])	28.15 (7.21)	30.18 (8.89)	27.98 (7.62)	F = 1.23; p = 0.281
HAMD, (mean [SD])	13.11 (4.14)	15.53 (5.54)	14.78 (4.48)	0.094
GAD-7, (mean [SD])	15.56 (3.27)	15;89 (2.88)	15.11 (3.17)	0.417
PSWQ, (mean [SD])	61;49 (7.08)	61.89 (7.95)	60.07 (8.67)	0.639
MCQ-30, (mean [SD])				
Total score	76.65 (14.46)	76.75 (15.20)	77.43 (13.28)	F = 0.18; p = 0.835
Factor 1	15.55 (5.23)	16.16 (5.06)	15.89 (5.89)	0.886
Factor 2	12.20 (4.96)	12.56 (4.81)	12.30 (4.13)	0.986
Factor 3	15.49 (4.17)	14.96 (3.68)	15.96 (3.62)	0.280
Factor 4	18.38 (3.74)	18.02 (4.42)	18.24 (4.23)	0.975
Factor 5	15.04 (3.73)	15.05 (3.81)	15.04 (3.55)	0.707

BMT = Body in Mind Training group; Factor 1 = cognitive confidence; Factor 2 = positive beliefs about worry; Factor 3 = cognitive self-consciousness; Factor 4 = negative beliefs about uncontrollability of thoughts and danger; Factor 5 = beliefs about the need to control thoughts; FLX = Fluoxetine group; GAD-7 = Generalized Anxiety Disorder 7-item scale; HAMA = Hamilton Anxiety Rating Scale; HAMD = Hamilton Rating Scale for Depression; MCQ-30 = Metacognitions Questionnaire; PSWQ = Penn State Worry Questionnaire; QoL = Quality of Life group; SD = standard deviation.

Values in bold indicate statistical significance.

* Estimated through chi-square test or Fisher’s exact test for categorical variables, analysis of variance (ANOVA) for continuous variables with normal distribution, and a nonparametric test for continuous variables with non-normal distribution.

### Scale validation

#### Reliability

The Brazilian version of the MCQ-30 scale had good internal consistency, as demonstrated by its Cronbach’s alpha coefficient of 0.89. All factors had results greater than or equal to 0.7 in this analysis: cognitive confidence had the highest result with 0.9; positive beliefs 0.86; cognitive self-consciousness 0.77; negative beliefs 0.83; and beliefs about the need to control thoughts 0.70. The MCQ-30 scale also has good reliability, as shown by the split-half test Spearman-Brown coefficient of 0.89.

#### EFA

Factor loadings are presented in Table 2 . Our data fitted the 5-factor model with a KMO of 0.83. Almost all items loaded onto a five-factor model, except for item 4, which loaded onto a sixth factor with a result of 0.99, and item 13, which loaded onto a seventh factor with a result of 0.50. This model explained 65.8% of the total variance.

**Table 2:** Factor loadings of MCQ-30 validation

	Factors				

	1	2	3	4	5
Cognitive confidence					
8. I have little confidence in my memory for words and names.	**0.79**	0.20	0.04	0.05	0.17
14. My memory can mislead me at times.	**0.77**	0.21	0.06	0.17	0.14
17. I have a poor memory.	**0.89**	0.19	-0.02	0.17	0.24
24. I have little confidence in my memory for places.	**0.60**	0.17	-0.01	0.15	0.21
26. I do not trust my memory.	**0.92**	0.21	-0.01	0.10	0.25
29. I have little confidence in my memory for actions.	**0.75**	0.21	-0.07	0.09	0.24
Positive beliefs about worry					
1. Worrying helps me to avoid problems in the future.	0.15	**0.54**	0.12	0.06	0.13
7. I need to worry in order to remain organized.	0.28	**0.65**	0.24	0.13	0.35
10. Worrying helps me to get things sorted out in my mind.	0.22	**0.86**	0.16	0.15	0.23
19. Worrying helps me cope.	0.15	**0.84**	0.29	0.08	0.33
23. Worrying helps me to solve problems.	0.21	**0.82**	0.28	0.12	0.24
28. I need to worry in order to work well.	0.12	**0.54**	0.27	0.17	0.35
Cognitive self-consciousness					
3. I think a lot about my thoughts.	0.07	0.13	0.38	**0.56**	0.45
5. I am aware of the way my mind works when I am thinking through a problem.	-0.09	0.02	**0.47**	0.13	0.27
12. I monitor my thoughts.	0.11	0.39	**0.63**	0.12	0.30
16. I am constantly aware of my thinking.	0.04	0.31	**0.69**	0.16	0.29
18. I pay close attention to the way my mind works.	-0.08	0.11	**0.83**	0.22	0.05
30. I constantly examine my thoughts.	0.03	0.17	**0.72**	0.39	0.39
Negative beliefs about uncontrollability and danger					
2. My worrying is dangerous for me.	0.06	0.08	0.19	**0.52**	0.46
4. I could make myself sick with worrying.*	0.08	0.11	0.26	0.44	0.37
9. My worrying thoughts persist, no matter how I try to stop them.	0.10	0.03	0.20	**0.85**	0.28
11. I cannot ignore my worrying thoughts.	0.08	0.09	0.21	**0.85**	0.43
15. My worrying could make me go mad.	0.23	0.07	0.12	**0.57**	0.54
21. When I start worrying I cannot stop.	0.16	0.14	0.18	**0.80**	0.41
Beliefs about the need to control thoughts					
6. If I did not control a worrying thought and then it happened, it would be my fault.	0.08	0.19	0.26	0.38	**0.61**
13. I should be in control of my thoughts all of the time.^†^	0.15	0.29	0.48	0.39	0.43
20. Not being able to control my thoughts is a sign of weakness.	0.23	0.25	0.27	0.41	**0.74**
22. I will be punished for not controlling certain thoughts.	0.19	0.19	0.19	0.31	**0.63**
25. It is bad to think certain thoughts.	0.15	0.12	0.27	**0.51**	0.40
27. If I could not control my thoughts, I would not be able to function.	0.13	0.23	0.39	0.21	0.23

MCQ-30 = Metacognitions Questionnaire.

Values in bold indicate the highest factor loading.

* Item 4 loaded onto factor 6 with 0.99.

^†^ Item 13 loaded onto factor 7 with 0.50.

Since item 4 loaded very powerfully (0.99) onto a different factor from in the original version, we decided to estimate an EFA excluding this item from the scale. Our results showed that all items loaded onto a five-factor model, like in the original scale,^19^ except for item 13, which loaded onto the cognitive self-consciousness factor (in contrast with the original version, in which it loads onto the beliefs about the need to control thoughts factor). All other items in this version loaded onto the same factors as in our 30-item EFA. However, this 29-item model of the scale explained only 59.8% of total variance, significantly lower than the 30-item model.

In our 30-item model, more than 80% of the items loaded onto the same factors as the original version and almost all items had loadings greater than 0.40 onto their corresponding factors, so we decided to keep the original format of 30 items in five subscales and their respective names. Items loaded onto the cognitive confidence and positive beliefs factors were the same as in the original version. Items 3 and 25 had the highest loadings onto a non-corresponding factor.

#### Confirmatory factor analysis (CFA)

We performed confirmatory analyses of the MCQ-30 using chi-square, RMSE, CFI, and TLI. Three out of the four goodness-of-fit tests used accepted the five-factor model. The chi-square test was significant (χ^2^ = 719.23, p-value < 0.01), which suggests that our data does not fit a five-factor model. However, the RMSEA result was 0.068 (90% confidence interval: 0.060-0.075), indicating an acceptable fit to the model, and CFI and TLI results were 0.87 and 0.86, respectively, which approximate to an acceptable fit to the model.

#### Convergent validity

The MCQ-30 and its subscales showed positive correlations with pathological worry. The MCQ-30 total score revealed a moderate correlation with the PSWQ scale ( *r* = 0.41, p < 0.01). The negative beliefs factor had the highest correlation ( *r* = 0.60, p < 0.01), while the need to control thoughts factor had a moderate correlation ( *r* = 0.42, p < 0.01) with the PSWQ. Positive beliefs and cognitive self-consciousness had small correlations ( *r* = 0.16, p < 0.05; *r* = 0.20, p < 0.01, respectively). Only the cognitive confidence factor had a poor and non-significant correlation with the PSWQ scale ( *r* = 0.03, p = 0.678).

## Efficacy analysis

Table 3 shows the model parameters from the GEE. We found a significant Group x Time interaction (p < 0.001); MCQ-30 scores decreased from baseline to week 8 in both the BMT (mean difference [MD] = -6.04, standard error [SE] = 2.39, p = 0.034) and the FLX (MD = -5.78, SE = 1.91, p = 0.007) groups. At the end of the treatment, FLX was superior to QoL (MD = -7.39, SE = 2.86, p = 0.03).

**Table 3:** - Model parameters from the GEE

	Estimated means* MD (SE)			Significance test* Wald χ^2^ (p-value)			Adjusted MD at week 8 MD, (95%CI), p-value*		
	
	BMT	FLX	QoL	Group	Time	Group x Time	BMT vs. QoL	FLX vs. BMT	FLX vs. QoL
MCQ-30									
Baseline	79.46 (2.71)	78.00 (3.09)	78.94 (2.76)	5.91	10.16	17.92	-6.19 (-12.98-0.60) 0.087	-1.20 (-8.29-5.91) 1.00	-7.39 (-14.24--0.53) **0.03**
Week 5	77.10 (2.72)	72.03 (3.10)	81.51 (3.59)	(0.052)	**(0.006)**	**(0.001)**			
Week 8	73.42 (2.95)	72.22 (3.33)	79.61 (3.00)						
Factor 1							-1.75 (-4.00-0.50) 0.189	0.26 (-1.86-2.39) 1.000	-1.49 (-0.50-4.01) 0.335
Baseline	15.83 (0.88)	15.95 (0.96)	15;61 (0.96)	2.35	4.35	12.89			
Week 5	14.56 (0.88)	14.90 (0.99)	16.65 (1.13)	(0.309)	(0.114)	**(0.012)**			
Week 8	14.50 (0.84)	14.76 (1.03)	16.25 (1.02)						
Factor 2							-2.43 (-4.62--0.25) **0.023**	1.98 (-0.13-4.08) 0.074	-0.46 (-2.50-1.58) 1.000
Baseline	12.39 (0.96)	12.67 (0.91)	12.55 (0.85)	2.27	4.98	15.54			
Week 5	13.18 (0.90)	12.58 (0.89)	13.95 (1.09)	(0.321)	(0.093)	**(0.004)**			
Week 8	11.78 (0.94)	13.76 (0.93)	14.22 (0.94)						
Factor 3							-0.33 (-2.24-1.57) 1.000	-1.42 (-3.28-0.43) 0.198	-1.75 (-3.54-0.03) 0.056
Baseline	15.79 (0.73)	15.02 (0.77)	16.12 (0.81)	6.63	1.97	2.49			
Week 5	16.83 (0.82)	15.07 (0.82)	16.33 (0.89)	**(0.036)**	(0.374)	(0.647)			
Week 8	16.36 (0.80)	14.94 (0.82)	16.70 (0.85)						
Factor 4							-0.79 (-3.09-1.50) 1.000	-1.44 (-3.61-0.72) 0.330	-2.24 (-4.33--0.14) **0.032**
Baseline	19.29 (0.58)	19.11 (0.67)	19.28 (0.67)	6.60	48.92	14.25			
Week 5	18.37 (0.75)	15.89 (0.72)	18.51 (0.78)	**(0.037)**	**(< 0.001)**	**(0.007)**			
Week 8	16.97 (0.79)	15.53 (0.73)	17.76 (0.79)						
Factor 5							-0.43 (-2.36-1.49) 1.000	-0.23 (-2.04-1.57) 1.000	-0.67 (-.256-1.22) 1.000
Baseline	15.60 (0.55)	15.07 (0.60)	15.20 (0.60)	2.37	21.58	5.41			
Week 5	13.57 (0.62)	13.66 (0.70)	16.68 (1.90)	(0.305)	**(< 0.001)**	(0.248)			
Week 8	13.76 (0.63)	13.53 (0.70)	14.19 (0.73)						

95%CI = 95% confidence interval; BMT = Body in Mind Training group; Factor 1 = cognitive confidence; Factor 2 = positive beliefs about worry; Factor 3 = cognitive self-consciousness; Factor 4 = negative beliefs about uncontrollability of thoughts and danger; Factor 5 = beliefs about the need to control thoughts; FLX = Fluoxetine group; GEE = generalized estimating equations;

MCQ-30 = Metacognitions Questionnaire; MD = mean difference; QoL = Quality of Life group; SE = standard error.

Values in bold indicate statistical significance.

* p-value for the comparison between the three intervention groups.

Considering the five factors of the MCQ-30 scale, we found a significant Group x Time interaction for cognitive confidence (p = 0.012), positive beliefs (p = 0.004), and negative beliefs (p = 0.007). Only the FLX group showed significant improvement in levels of cognitive confidence from baseline to week 8 (MD = -1.18, SE = 0.48, p = 0.041). At the end of the treatment, BMT was superior to QoL (MD = -2.43, SE = 0.91, p = 0.023), while there was no difference in the comparisons of BMT with FLX or FLX with QoL.

Subscale-rating scores for negative beliefs about uncontrollability and danger decreased over time in the BMT (MD = -2.32, SE = 0.71, p = 0.003), FLX (MD = -3.59, SE = 0.54, p < 0.001), and QoL (MD = -1.52, SE = 0.58, p = 0.026) groups. At week 5, FLX was superior to QoL (MD = -1.18, SE = - 0.39, p = 0.008). Similarly, at week 8, FLX was superior to QoL (MD = -2.24, SE = 0.87, p = 0.032), but not to BMT, and there was no difference between BMT and QoL. Scores for beliefs about the need to control thoughts decreased over time in both the BMT (MD = -1.84, SE = 0.64, p = 0.012) and FLX (MD = -1.54, SE = 0.50, p = 0.007) groups.

### Correlation analysis

**Figure 1:**
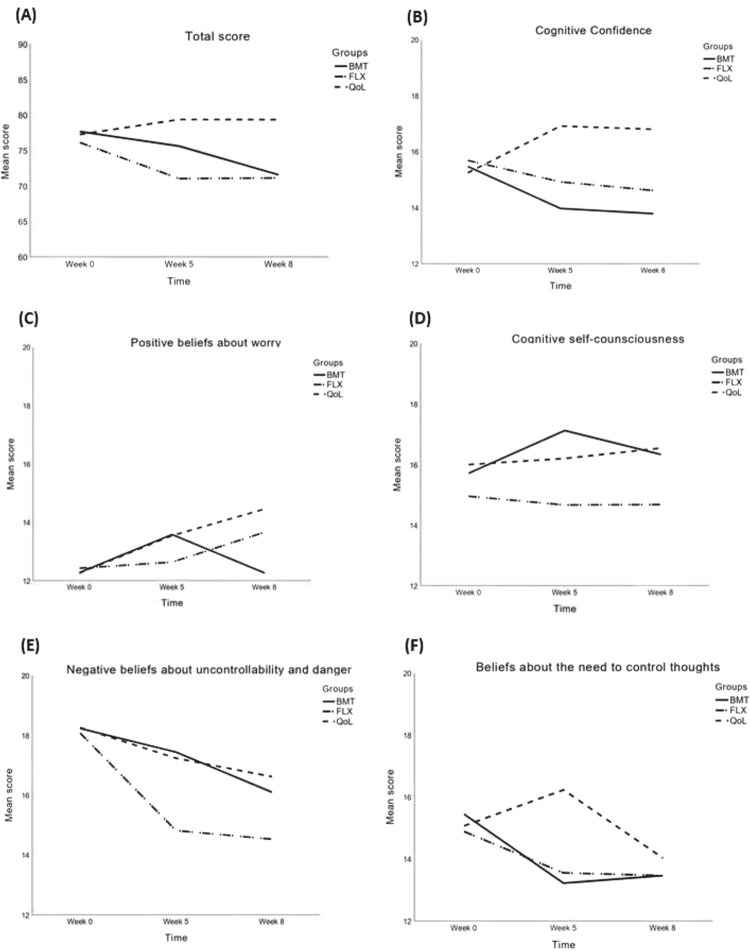
Mean scores for the MCQ-30 and its five subscales at baseline, week 5, and week 8 of the interventions. BMT = Body in Mind Training; FLX = Fluoxetine; MCQ-30 = Metacognitions Questionnaire; QoL = Quality of Life.

The results for differences in scores from baseline to endpoint of the intervention can be found in Table 4 and Figure 1 . We found a fair positive correlation between the mean change (delta) in PSWQ and MCQ-30 from baseline to the endpoint (Rs = 0.477, p < 0.001). When analyzing each of the intervention groups separately, there was a positive correlation between these scores in the BMT (Rs = 0.540, p = 0.009) and FLX (Rs = 0.550, p < 0.001) groups; there was no significant correlation between the deltas of the scales in the QoL group (Rs = 0.289, p = 0.128). There was also a fair positive correlation between changes in the subscale negative beliefs about worry and the PSWQ scale (R = 0.535, p < 0.001).

**Table 4:** Differences in scores from baseline to endpoint of the intervention

Scales	Mean difference Δ (SE)*	p-value	Effect size of differences d (95%CI)
MCQ-30 total			
BMT	-6.04 (2.39)	**0.034**	-0.42 (-0.78--0.05)
FLX	-5.78 (1.91)	**0.007**	-0.46 (-0.83--0.10)
QoL	0.67 (1.70)	1.000	-
Factor 1			
BMT	-1.33 (0.62)	0.094	-0.42 (-0.79--0.06)
FLX	-1.18 (0.48)	**0.041**	-0.44 (-0.81--0.08)
QoL	0.64 (0.49)	0.569	-
Factor 2			
BMT	-0.61 (0.67)	1.000	-0.26 (-0.62- 0.10)
FLX	1.09 (0.55)	0.146	0.10 (-0.26- 0.46)
QoL	1.66 (0.59)	**0.008**	-
Factor 3			
BMT	0.57 (0.56)	0.919	-0.002 (-0.36-0.36)
FLX	-0.08 (0.50)	1.000	-0.174 (-0.53- 0.19)
QoL	0.58 (0.49)	0.693	-
Factor 4			
BMT	-2.32 (0.71)	**0.003**	-0.16 (-0.52- 0.20)
FLX	-3.59 (0.54)	**< 0.001**	-0.48 (-0.85--0.12)
QoL	-1.52 (0.58)	**0.026**	-
Factor 5			
BMT	-1.84 (0.64)	**0.012**	-0.19 (-0.55- 0.17)
FLX	-1.54 (0.50)	**0.007**	-0.14 (-0.50- 0.22)
QoL	-1.00 (0.49)	0.121	-

95%CI = 95% confidence interval; BMT = Body in Mind Training group; Factor 1 = cognitive confidence; Factor 2 = positive beliefs about worry; Factor 3 = cognitive self-consciousness; Factor 4 = negative beliefs about uncontrollability of thoughts and danger; Factor 5 = beliefs about the need to control thoughts; FLX = Fluoxetine group; MCQ-30 = Metacognitions Questionnaire; QoL = Quality of Life group; SE = standard error.

Values in bold indicate statistical significance.

* Adjusted for obsessive-compulsive disorder diagnosis.

## Discussion

This study aimed to evaluate the reliability, internal consistency, construct validity, and convergent validity of the MCQ-30 scale in Brazilian patients with GAD and took advantage of the gold standard design to also assess the interventions’ efficacy for reducing metacognitive beliefs, considering three different interventions (BMT, FLX, and QoL).

### Scale validation

Our study showed that the MCQ-30 scale has good psychometric properties, allowing its use in clinical settings and in further studies with Brazilian populations. Overall, acceptable internal consistency, reliability and validity were demonstrated. The CFA supports the original five-factor model proposed by Wells.^22^ However, the EFA generated a seven-factor model with 30 items and a five-factor model excluding item 4 from the scale. Since the 29-item model explained a significantly lower proportion of the total variance that the 30-item model (65.8% for the 30-item model vs. 59.8 for the 29-item model), we decided to maintain item 4. It is possible that item 4 generated a new factor with such impact in EFA because of differences in content validity between the two languages: whereas “sick” in English means “affected by physical or mental illness”^30^ or “feeling nauseous and wanting to vomit,”^30^ which is a more reliable physical response to anxiety, the word used for “sick” in the Portuguese translation only means “being affected by physical or mental illness.”^31^ We decided to maintain a five-factor structure containing the original items, since more than 80% of the items loaded onto the original factors.

When comparing the present study to validation studies performed in Spain^32^ and Serbia,^33^ our sample exhibited significantly higher scores on the MCQ-30 scale. It is possible that cultural differences between these populations and differences in patient selection may contribute to this finding. Our sample consisted of patients diagnosed with GAD, whereas the studies by Martín et al.^32^ and Markovic et al.^33^ included patients with various anxiety and depression disorders. Moreover, in accordance with the studies mentioned above, the subscale negative beliefs about uncontrollability and danger had the highest score of the five subscales, supporting previous results that negative beliefs about worry is the most characteristic domain of metacognition in GAD.^8^ Additionally, these two studies and ours are in accordance with the positive correlation between the MCQ-30 and levels of pathological worry, as evidenced by its convergent validity with the PSWQ scale. More specifically, the negative beliefs about uncontrollability and danger factor showed the highest correlation (r = 0.60, p < 0.01). These results are in agreement with Wells’ hypotheses^11^ concerning the cardinal role played by negative beliefs in the development of anxiety through a process of meta-worry.

Despite the strengths of this study to validate the MCQ-30 for Brazilian samples, some limitations should be noted. First, the study sample was predominantly young, female, and from economic classes B and C and 71.3% of participants had at least incomplete higher education. It may not therefore have been representative of the whole Brazilian population. The recruitment process using social media can partially explain the characteristics of the present population. Furthermore, GAD is known to be more prevalent in the female gender and in high-income countries.^34^ Second *,* since the sample was not originally recruited for a validation study, we had a smaller number of participants than is recommended for the item-to-participant validation process, i.e. a 1:10 ratio.^35 , 36^ This small sample size could explain the acceptable but suboptimal model fit observed in our study, as evidenced by our CFA results. Finally *,* only participants diagnosed with GAD were included in our sample. Future studies involving the MCQ-30 should include non-clinical samples and samples with other disorders, such as depression for example.

### Efficacy analysis

Both BMT and FLX were able to reduce MCQ-30 scores from baseline to endpoint. However, only the FLX group was superior to QoL at week 8, and there was no statistically significant difference between FLX and BMT. When analyzing each of the MCQ-30 subscales, we found different results. There were no differences in the cognitive confidence factor scores between the three groups, and only FLX improved them from baseline to the endpoint. Furthermore, we did not find significant differences in scores for the positive beliefs about worry factor. Therefore, these interventions were unlikely to reduce anxiety by intervening in lack of cognitive confidence or by reducing positive beliefs about worry. None of the interventions showed modifications in scores for the cognitive self-consciousness factor over time. Thus, perhaps pharmacological treatment and MBIs do not prevent thought examination. Rather, they may reduce anxiety by enabling more flexible metacognition, in which patients remain aware of their own thoughts, but do not react excessively to them.

On the other hand, the performance of the MCQ-30 negative beliefs factor was more consistent than the other factors, which corroborates the literature suggesting that this factor is a core characteristic of GAD^8^ and has a positive correlation with anxiety symptoms.^9^ Although all intervention groups showed statistically significant improvement from baseline to the endpoint, FLX was superior when compared to the QoL group and showed no difference when compared to BMT. Finally, there was no significant Group x Time interaction for the beliefs about the need to control thoughts factor, but this subscale rating score improved from baseline to week 8 in the BMT and FLX groups. This finding reinforces the potential effect on cognitive processes related to GAD of the two interventions mentioned above.

To our knowledge, there are no data on changes in metacognition after pharmacological treatment. Considering that in our study FLX was the most effective intervention for reducing anxiety levels, which was the main outcome of the clinical trial,^17^ our first hypothesis was that pharmacotherapy reduced metacognitive beliefs through improvement in worry itself. The improvement in anxiety symptoms would reduce the association between worry and anxiety and, therefore, reduce the levels of negative beliefs about worry and the adoption of thought control and other avoidant strategies. A study by Capobianco et al.^37^ demonstrated that, just as metacognitive beliefs predict development of anxiety, levels of anxiety predict later metacognition. Interestingly, our correlation analysis showed a positive association between changes in MCQ and PSWQ from baseline to the endpoint of the trial in the FLX and BMT groups, and, in accordance with Wells et al.,^6^ the reduction in the negative beliefs about worry subscale was correlated with improvement in worry. Although a possible effect of FLX on metacognition was observed, we do not yet know whether this effect persists after discontinuation of pharmacotherapy for GAD.

Previous studies showed that MBIs are able to reduce metacognitive beliefs.^38 , 39^ McEvoy et al.^38^ compared Mindfulness-Based Progressive Muscle Relaxation to Metacognitive Therapy and a control group in individuals with high trait anxiety, while a meta-analysis by Rogers et al.^39^ included use of different MBIs in overweight patients and both studies found that MBI reduced metacognitive beliefs. However, these studies analyzed samples with different diagnoses and used MBI protocols other than BMT. Another possible reason for the inconsistent results found in the BMT group could be a lack of patient adherence, since this intervention required daily practice of mindfulness techniques at home, and we did not assess patient compliance outside the trial environment. Also, the short duration of the intervention and the absence of long-term follow-up may have played a role, considering that some important meaningful effects on metacognition may only appear after some time of treatment.

Our results need to be interpreted with some caution since they derive from a sub-analysis of a clinical trial. Among other limitations, we may cite the relatively short follow-up time, which may have underestimated the effect of the pharmacological treatment. Nevertheless, this study offers new insight into the metacognitive model of GAD and suggests that metacognition may be a potential therapeutic target in GAD. The MCQ-30 scale, in particular its negative beliefs about uncontrollability and danger factor, could be employed to verify treatment response to psychotherapy or pharmacological treatment. Further longitudinal studies with larger samples, long-term follow up, and greater power to detect differences in metacognitive beliefs should be conducted to clarify the effect on metacognition of different interventions for GAD.

## Conclusion

Dysfunctional metacognitive beliefs are an important characteristic in the development and maintenance of GAD.^6^ Our study is the first to validate the MCQ-30 scale in a Brazilian sample and to assess possible effects on metacognitive beliefs of different interventions for GAD. Overall, the MCQ-30 showed good psychometric properties in our sample. Future studies must validate MCQ-30 in Brazilian samples other than people with GAD. Moreover, we demonstrated a possible effect of FLX and BMT on metacognitive beliefs, especially on negative beliefs about uncontrollability and danger. Therefore, further investigations are necessary to clarify the potential benefits of targeting metacognitive beliefs in treatment of patients with GAD.
